# GATA Transcription Factors: A Cross‐Road for Erythropoiesis, Neurodevelopment, and Synucleinopathies

**DOI:** 10.1002/dneu.22975

**Published:** 2025-06-06

**Authors:** Francesco Bellomi, Claudia Caturano, Viola Velardi, Romina Mancinelli, Simone Carotti, Giorgio Vivacqua, Francesca Arciprete, Maria Zingariello

**Affiliations:** ^1^ Laboratory of Microscopic and Ultrastructural Anatomy Campus Bio‐Medico University of Roma Roma Italy; ^2^ Section of Human Anatomy, Department of Anatomic, Histologic, Forensic Medicine and Locomotor Apparatus Sciences Sapienza University of Roma Roma Italy

**Keywords:** erythropoiesis, GATA transcription factors, neuroanatomy, Parkinson's disease, α‐synuclein

## Abstract

Alpha‐synuclein (α‐syn), a 140 amino acid protein, is abundantly expressed in the central nervous system (CNS) and in the erythrocytes, playing a pivotal role in the pathogenesis of Parkinson's disease (PD) and other synucleinopathies. Among the GATA family transcription factors (TFs), GATA1 and GATA2 regulate the meg‐erythrocytic differentiation starting from the hematopoietic stem cell. In erythropoiesis, the GATA1‐2 switching regulates the expression of the α‐syn gene (SNCA) in the erythrocytes, which is essential for iron metabolism and membrane stability. Abnormalities in α‐syn regulation alter erythrocytic function, possibly contributing to pathological mechanisms of different synucleinopathies. In CNS, during neuronal development, GATA2 confirms its role in stemness by maintaining the ventral neuronal progenitors and also leading GABAergic, serotoninergic, and sympathetic neuron differentiation. Therefore, although no evidence is reported regarding a direct role of GATA1 in neuronal lineage, GATA3 recruitment and activation are essential for the maturation of specific neuronal subtypes. This short scope review explores the bridging role of GATA TFs in erythropoiesis and neuronal development, highlighting their involvement in α‐syn regulation, as well as their potential role in the pathogenesis of synucleinopathies.

**Abbreviations**
9‐me‐BC: 9‐methyl‐β‐carboline
AGM: aorta–gonad–mesonephros region
ALAS2: delta‐aminolevulinate synthase 2
BasoE: basophilic erythroblast
BBB: blood brain barrier
BFU: burst‐forming unit erythroid
BLVRB: biliverdin reductase B
CFU‐E: colony‐forming unit erythroid
ChIP: chromatin immunoprecipitation
CNS: central nervous system
CREB1: CAMP responsive element binding protein 1
CREBBP: CREB binding protein
DG: dentate gyrus
DLB: dementia with Lewy bodies
DMRT1: doublesex and mab‐3 related transcription factor 1
dpc: days post coitum
EKLF: erythroid Krüppel‐like factor
EPO: erythropoietin
EVs: extracellular vesicles
FECH: ferrochelatase
FOG1: friend of GATA1
G1HRD: GATA1 hematopoietic regulatory domain
GATA‐bHLH‐BNR: GATA–basic/helix–loop–helix–BNR
GPi: globus pallidus internus
Hgb: hemoglobin
HOXA13: homeobox A13
HOXB13: homeobox B13
HOXC13: homeobox C13
HOXD13: homeobox D13
HSC: hematopoietic stem cells
iNOS: inducible nitric oxide synthase
KLF1: Krüppel‐like factor 1
KO: knock out
LRRK2: leucine‐rich repeat kinase 2
MEP: megakaryocyte–erythroid progenitor
MPTP: 1‐methyl‐4‐phenyl‐1,2,3,6‐tetrahydropyridine
MSA: multiple system atrophy
MVE: multivesicular endosomal
NA: noradrenaline
NCSC: neural crest stem cell
NFE2: nuclear factor erythroid 2
Nkx2‐2: Nk2 homeobox 2
OrthoE: orthochromatic erythroblast
PD: Parkinson's disease
Pitx3: paired like homeodomain 3
PolyE: polychromatophilic erythroblast
POU4F1: POU class 4 homeobox 1
POU4F2: POU class 4 homeobox 2
POU4F3: POU class 4 homeobox 3
ProE: proerythroblast
R1: rhombomere 1
R2: rhombomere 2
R4: rhombomere 4
RBC: red blood cell
RBPJ: recombination signal binding protein for immunoglobulin kappa j region
ROS: reactive oxygen species
Sert: serotonin transporter
SMA: spinal muscular atrophy
SN: substantia nigra
SNAI2: snail family transcriptional repressor 2
SNAI3: snail family transcriptional repressor 3
SRF: serum response factor
SSR: simple sequence repeat
SUV: small unilamellar vesicles
SVZ: sub‐ventricular zone
TCF3: T cell factor 3
Tf/TfR: transferrin/transferrin receptor
TfR1: transferrin receptor‐1
TFs: transcription factors
TH: tyrosine hydroxylase
Vglut3: vesicular glutamate transporter 3
VTA: ventral tegmental area
WT1: WT1 transcription factor
α2ϵ2: embryonic hemoglobin Hb gower II
α‐syn: alpha‐synuclein

## 1 Introduction

Alpha‐synuclein (α‐syn) and GATA transcription factors (TFs) play a pivotal bridging role in cell biology, with significant implications for both erythropoiesis and neuronal function and neuronal development (Abad et al. 2024; Cheng et al. 2011).

α‐Syn is a 140 amino acid protein, richly expressed in the central and peripheral nervous system, where it has been detected at presynaptic terminals and in neuronal cell nuclei, with a differential expression in distinct brain territories (Bordbar et al. 2024; Jakes et al. 1994; Vivacqua et al. 2009). Though its complete physiological role remains to be fully elucidated, substantial evidence supports the involvement of α‐syn in synaptic function and plasticity, as well as neurotransmitter release (Cheng et al. 2011; Sharma and Burré 2023). α‐Syn is the key pathogenetic protein in synucleinopathies, a group of neurodegenerative disorders that includes Parkinson's disease (PD). In these pathological contexts, α‐syn aggregates to form Lewy bodies and Lewy neurites—the signature pathological markers of PD and dementia with Lewy bodies (DLB) (Spillantini et al. 1997; Tofaris and Spillantini 2005)—or glial and nuclear cytoplasmic inclusions, as pathological hallmarks of multiple system atrophy (MSA) (Reddy and Dieriks 2022). The misfolding and aggregation of α‐syn disrupt normal neuronal signaling, leading to synaptic dysfunction and SNARE complex re‐organization (Brolin et al. 2023; Burré et al. 2014; Faustini et al. 2018; Garcia‐Reitböck et al. 2010; Serra et al. 2025), microglial alterations (Eo et al. 2024; Pei et al. 2024), and mitochondrial stress (Eo et al. 2024; Gąssowska‐Dobrowolska et al. 2024; Geibl et al. 2024). Furthermore, recent evidence suggests that α‐syn plays a pivotal role in gene regulation and transcription in both neurons (Baptista et al. 2003; Li et al. 2011; Schneider et al. 2007; Sugeno et al. 2016) and erythroid lineage (He et al. 2024; Ling et al. 2022), underscoring the necessity of exploring not only its pathological implications but also its regulatory functions in cell biology and gene expression.

GATA TFs are a family of regulatory proteins characterized by their highly conserved DNA‐binding domain. They are master regulators of gene expression during various developmental processes, particularly in hematopoiesis and cardiac development (Grunert et al. 2024; Liao and Bresnick 2024). Different GATA TFs play distinct yet overlapping roles in the differentiation and maturation of different cell lines, regulating the transcription of specific genes involved in the process of differentiation. For example, by modulating the expression of genes necessary for the survival and proliferation of erythroid progenitors, GATA factors ensure proper red blood cell (RBC) formation, adequate oxygen transport, and maintenance of systemic oxygen homeostasis (Tang and Wang 2023). Intriguingly, emerging research suggests a connection between GATA TFs and α‐syn (Scherzer et al. 2008), highlighting the broader implications of both proteins either in erythropoiesis or in central nervous system (CNS) functions and development. For instance, studies suggest that α‐syn may influence the expression of GATA TFs, thereby affecting hematopoietic processes (Naushad et al. 2021). However, GATA factors may exert regulatory control over genes implicated in α‐syn expression and function (Afek et al. 2018), creating a complex molecular loop between hemopoietic and neural pathways and revealing a fascinating intersection of peripheral and central functions.

Particularly, the bridging role of α‐syn and GATA TFs underscores a dynamic interdependence that may impact not only the maturation of the RBCs but also the integrity and functionality of the CNS, with implications for neurodevelopment and neurodegeneration. Therefore, in this review, we explore this intertwined molecular connection, paving new possible biological mechanisms for the pathogenesis of synucleinopathies.

## 2 Biological Bases of Erythropoiesis

Erythropoiesis first occurs in the yolk sac (Kinder et al. 1999; Lawson et al. 1991). The mesoderm/endoderm interaction in the yolk sac generates the blood islands, which constitute the first input of erythropoiesis (Belaoussoff et al. 1998; Dzierzak and Speck 2008) both in mice and human embryos (Bernstein 1996; Tavian et al. 1999). The progenitor cells in the blood islands differentiate into erythroblasts, which produce embryonic hemoglobin (Hb) (α2ϵ2) (Yamane 2020). The human embryonic erythropoiesis shifts from the yolk sac to the liver and lastly to the bone marrow, where hematopoiesis takes place after birth (Tang and Wang 2023). In adults, erythroid differentiation originates from hematopoietic stem cells (HSCs), which originate—in the embryo—at a specific portion of the ventral mesoderm of the abdominal aorta, termed aorta–gonad–mesonephros (AGM) region (Dzierzak and Speck 2008). The AGM cells that promote erythropoiesis derive from specialized endothelial cells with a “hemogenic” phenotype (Dieterlen‐Lièvre and Martin 1981). The fact that HSCs originate from functioning vascular endothelial cells was experimentally confirmed by the Robin laboratory showed the endothelial‐to‐hematopoietic cell transition in mid‐gestation mouse aorta (Boisset et al. 2010).

In the adult bone marrow, the HSC starts its differentiation in the common myeloid progenitor. The first erythroid progenitor, originating from the megakaryocyte–erythroid progenitor (MEP) cells, is the burst‐forming unit erythroid (BFU‐E), which in turn differentiates into the late progenitor colony‐forming unit erythroid (CFU‐E). Subsequently, CFU‐E become increasingly dependent on erythropoietin (EPO) and undergo terminal differentiation (Zhang et al. 2012).

**FIGURE 1:**
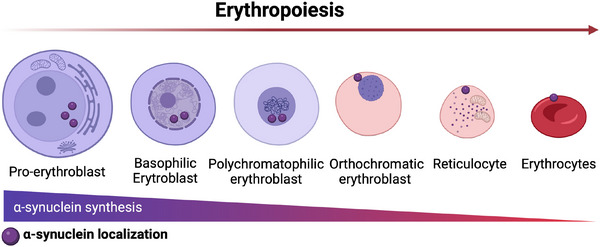
**Erythropoiesis**. The process of human erythropoiesis begins with early erythropoiesis and concludes with the maturation of reticulocytes. During terminal erythroid differentiation, the pro‐erythroblast emerges as the first morphologically recognizable erythroid precursor, characterized by a large nucleus with nucleoli and free ribosomes in the cytoplasm. Beta‐globin synthesis begins at this stage. Basophilic erythroblasts and polychromatophilic erythroblasts exhibit increasing heterochromatin and mitotic activity. In orthochromatic erythroblasts, the nucleus condenses and is expelled, forming reticulocytes that lack a nucleus but retain residual RNA. Reticulocytes mature into biconcave, deformable erythrocytes devoid of organelles and specialized for oxygen transport. The location of α‐synuclein change within the cell changes during erythroid development. It is mainly found in the nucleus of erythroblasts, but as erythropoiesis progresses, it moves to the plasma membrane in reticulocytes and erythrocytes. *Source*: Created in BioRender (https://BioRender.com/b47z219).

The human erythropoiesis process starts from the early erythropoiesis to the terminal erythroid differentiation and reticulocyte maturation, as summarized in Figure 1 (Huang et al. 2020).

Upon the terminal erythroid differentiation phase, the first morphologically recognizable erythrocytic precursor is the pro‐erythroblast (ProE). This cell (20–25 µm in diameter) is characterized by a large rounded nucleus with visible nucleoli. Free ribosomes are dispersed into the cytoplasm. Morphological changes in the basophilic erythroblast (BasoE) and in the polychromatophilic erythroblast (PolyE) lead to the increased heterochromatin and mitotic activity. During erythrocytic maturation, a progressive accumulation of Hb occurs in the erythroblasts. This results in a progressive conversion of the erythrocytic cytoplasm from basophilic to polychromatophilic (Breton‐Gorius and Reyes 1976). In the PolyE cells, the nuclear size is progressively smaller, with coarse heterochromatin granules. In the last phases of the erythroid differentiation, the orthochromatic erythroblast (OrthoE) has a highly condensed nucleus (Popova et al. 2009) and an almost completely acidophilic cytoplasm because of the increased Hgb amount. In this cell, the mitotic division is arrested; the significant change in the morphology is due to the nuclear extrusion, whereas the polyribosomes are clumped and arranged in a reticular network. The neo‐formed reticulocyte lacks the nucleus but has residual RNA and a limited ability for transporting oxygen (Caulier and Sankaran 2022; Dzierzak and Philipsen 2013). The mature erythrocyte (7–8 µm) lacks both a nucleus and organelles. The organelle elimination, as well as morphological changes occur during the reticulocyte maturation. The reticulocyte is a multilobulated cell (Mel et al. 1977). Upon maturation, it loses volume and surface area; thanks to the cytoskeletal rearrangement, it assumes the shape of a biconcave disk and becomes a deformable cell (Waugh et al. 2001, 1997). Moreover, the changes in the expression of many membrane proteins are related to the membrane remodeling. The multivesicular endosomal (MVE) pathway was first described for the transferrin receptor‐1 (TfR1) regulation, and it constitutes a membrane protein involved in Hb synthesis (Harding et al. 1983; Pan and Johnstone 1983). Together with the autophagy (Ney 2011; Tanaka 1970), the MVE is essential for the organelles’ clearance, because the MVE terminal steps result in the release of intraluminal vesicles, the exosomes, into the extracellular space.

Erythroid differentiation occurs in response to specific stimuli, including cytokines, TFs (GATA1, erythroid *Kruppel‐like factor* [*EKLF*]/KLF1, NFE2, and SCL), and cofactors (friend of GATA1 [FOG1], *T cell factor 3* [TCF3], CBP/p300, TRAP200, BRG1), as well as other signal transduction proteins that cooperate with each other and therefore are defined as lineage restricted (Kim and Bresnick 2007; Doré and Crispino 2011; Tsiftsoglou et al. 2009).

The TF GATA1 is required for the terminal differentiation of MEP cells into definitive erythroid cell lineage (Papetti et al. 2010). GATA1 promotes cell survival through the activation of antiapoptotic genes of the BCL family and promotes the EPO receptor expression (Zon et al. 1991). Under GATA1 influence, the MEP cells transform into EPO‐sensitive erythrocyte‐committed progenitors that give rise to ProE. At this stage, the cell maintains its nucleus until its maturation. The next stepwise lead to the differentiation in normoblasts, when the nucleus is ejected from the cell. Moreover, *FOXO3* regulates the different maturation phases of the erythroid cell and, in the late phase, participates in the expression of the genes responsible for cell enucleation. In the terminal erythroid differentiation after the enucleation, in the neo‐differentiated reticulocyte, GATA1 activates expression of erythroid‐specific genes, such as α‐ and b‐ globin, heme biosynthesis enzymes, and erythroid membrane proteins (Lally et al. 2019).

In erythrocytes, the gene encoding for α‐syn—*SNCA*—is transcribed in a co‐expression with the heme metabolism genes delta‐aminolevulinate synthase 2 (*ALAS2*), ferrochelatase (*FECH*), and biliverdin reductase B (*BLVRB*), all regulated by the GATA1 TF (Scherzer et al. 2008). α‐Syn has been reported to have a crucial role in erythropoiesis and the lifespan of RBCs by regulating membrane curvature, a fundamental aspect also shared with synaptic vesicle dynamics. This protein is highly expressed in RBCs, where it contributes to maintaining their biconcave shape, essential for flexibility and functionality in oxygen transport. Recent studies have highlighted that the accumulation of aggregated α‐syn on RBC membranes is associated with an increase in acanthocytic cells and compromised membrane integrity, phenomena observed in murine models and patients with neurodegenerative diseases such as PD (Shin et al. 2000; Yang, Shi, et al. 2024) and neuroacanthocytosis (Feriante and Gupta 2025; Schon et al. 2024). Furthermore, α‐syn acts by reducing methemoglobin to functional Hb through its ferrireductase capacity, underscoring its contribution to RBC survival by preventing oxidative damage (Kuo and Nussbaum 2015). α‐Syn interacts with phospholipid membranes, displaying a preference for curved structures such as small unilamellar vesicles (SUVs). The N‐terminal acetylation of α‐syn stabilizes its helical conformation, reducing the entropic cost of membrane binding and enhancing interaction with lipid bilayers. This acetylation influences binding affinity to specific lipids, particularly phosphatidylserine, and modulates its aggregation propensity into fibrils, suggesting that α‐syn membrane‐binding properties may be crucial for maintaining RBC membrane stability and morphology (Iyer et al. 2016).

## 3 GATA TFs in the Nervous System

Growing evidence highlights the involvement of the GATA TFs family in the brain and nervous tissue (Katsumura and Bresnick 2017). This emerging role encompasses critical processes in neural differentiation and the maintenance of nervous system functionality.

In contrast to GATA1, for which there is currently no evidence of expression in the brain, TFs like GATA2 and GATA3, in addition to skin (Kaufman et al. 2003), lens (Maeda et al. 2009), and mammary gland (Asselin‐Labat et al. 2007; Moriguchi et al. 2018), are essential for progenitor cell maintenance and lineage specification in neuroectoderm‐derived tissues (Fujiwara 2017; Rodrigues et al. 2012). In the CNS, GATA2 and GATA3 perform overlapping and interdependent functions during the early stages of development (Tsai et al. 1994; Nardelli et al. 1999). In particular, *GATA2* not only regulates its own expression but also activates *GATA3*, thereby facilitating neuronal differentiation and ensuring the maintenance of ventral neuronal progenitors during early embryogenesis (Nardelli et al. 1999). Interestingly, the study conducted by Tremblay et al. (2018) demonstrates how *GATA2* is involved in the development of GABAergic and sympathetic neurons, whereas *GATA3* regulates the expression of tyrosine hydroxylase (TH), an enzyme essential for catecholamine biosynthesis, within sympathetic neurons. Therefore, according to researchers’ findings, the loss of *GATA3* in mice results in noradrenaline (NA) deficiency and embryonic lethality, which can be rescued with catecholamine intermediates.

During early development, GATA2 is thought to be fundamental in maintaining the pool of ventral neuronal progenitors, with its loss resulting in significant defects in neurogenesis (Tremblay et al. 2018). In contrast, Wakil's group revealed a new aspect of the function of GATA2, showing that it negatively regulates the proliferation of neuronal progenitors during neurogenesis (El Wakil et al. 2006). The study on mouse and chick embryos shows that GATA2 could inhibit Notch signaling, which caused the arrest of proliferation and the induction of neuronal differentiation. In mouse neuroepithelial progenitors, inactivation of GATA2 increases proliferation, whereas its overexpression arrests cell division without necessarily inducing neuronal differentiation (El Wakil et al. 2006). Moreover, current investigations have indicated that GATA2, due to its presence in regions where GABAergic neurons are produced (Xiong et al. 2024), plays a role in the development of GABAergic neuron identity in the midbrain and serotonergic neuron development in rhombomere 1 (r1). In this context, it functions as a post‐mitotic cell‐fate selector gene, a role similar to that of other TFs such as Ptf1a, which is involved in determining GABAergic identity in the cerebellum and spinal cord (Kala et al. 2009). Furthermore, Lahti et al. (2015) have shown that different GABAergic and glutamatergic subgroups associated with the monoaminergic nuclei express specific TFs. These neurons share common origins in the ventrolateral r1, where the post‐mitotic selector genes *Tal1*, *Gata2*, and *Gata3* control the balance between the generation of inhibitory and excitatory neurons. In the absence of Tal1, or both GATA2 and GATA3, the GABAergic precursors adopt glutamatergic fates and populate the glutamatergic nuclei in excessive numbers (Lahti et al. 2015). Moreover, in the work proposed by Nardelli et al., GATA2 was detected as early as 9 days post coitum (dpc) in the hindbrain, particularly in ventral rhombomere 4 (r4) and transiently in rhombomere 2 (r2). Between 9.5 and 11.5 dpc, the expression pattern expands to areas of early neuronal differentiation, including the olfactory bulbs, pretectum, and oculomotor nucleus of the midbrain. Likewise, GATA2 also extends to a floor plate stripe from the mesencephalon to the cervical spinal cord, as well as to the ventral columns containing motor neuron and interneuron precursors, and both the ventricular and subventricular zones of the neural tube (Nardelli et al. 1999). Notwithstanding the well‐established GATA2 expression across various mammalian tissues (Rubel et al. 2012), including neural (El Wakil et al. 2006), hematopoietic (Vicente et al. 2012), cardiovascular (Connelly et al. 2006), and urogenital systems (Khandekar et al. 2004), its expression in the mouse midbrain has been demonstrated to be regulated by two identified distinct domains: the distal 5H and the proximal 2H domains, located 3.0 and 1.9 kbp upstream of the transcriptional start site, respectively, both containing GATA factor binding sites (Nozawa et al. 2009). Although further studies are needed to fully elucidate the physiological contribution of GATA2 to the midbrain formation and function, the study conducted by Nozawa et al. demonstrated that both the 2H and 5H domains can independently activate *GATA2* gene expression in the superior colliculus of the midbrain, whereas the distal‐5H domain is capable of initiating GATA2 transcription in the inferior colliculus. These findings emphasize the pivotal function of these domains in regulating GATA2 expression in the midbrain and underscore the positive autoregulatory mechanism that governs *GATA2* transcription in nervous tissue (Nozawa et al. 2009).

Conversely, GATA3 is primarily confined to the subventricular zone of the neuronal tube and, interestingly, shows delayed activation compared to GATA2 (Muroyama et al. 2005). It is noteworthy that in *GATA2* knockout embryos, GATA3 expression in the CNS is severely impaired, indicating that *GATA2* is essential for the activation of GATA3 during early development (Nardelli et al. 1999).

Emerging evidence has suggested the presence and the expression of GATA3 TF in neurons of the adult pretectal region and tectum, regions associated with the control of the intrinsic and extrinsic eye movements (Kornhauser et al. 1994). These include visual and auditory input‐induced motor reflexes, as well as the transmission of sensory information related to eye movements to higher telencephalic regions. GATA3 expression has also been demonstrated in some GABAergic neurons and other neuronal subtypes of the *substantia nigra* pars reticulata, important for the modulation of dopaminergic neurons in the *pars compacta* and as an output nucleus of the circuit of the basal ganglia, together with the *globus pallidus internus* (GPi) (Zhao et al. 2008). Recent findings indicate that GATA3 may exert an influence also on NA neuron development. This hypothesis is supported by observations that increased GATA3 expression leads to a higher number of TH‐expressing neurons in primary neural crest stem cell (NCSC) cultures (Hong et al. 2006). The dynamic and region‐specific expression pattern of GATA3 in the CNS during embryonic development underscores a pivotal role in several CNS developmental stages (Kornhauser et al. 1994). For instance, as illustrated by George et al., although initially, during early embryogenesis, GATA3 expression is low, it then, later in gestation, starts becoming increasingly localized to specific CNS regions, including the mesencephalon (midbrain), diencephalon, pons, and spinal cord. In the spinal cord, during the early stages of development (before embryonic Day 10, e10), GATA3 expression is widespread and diffuse. By e12.5, its expression becomes restricted to the ventral region of the spinal cord, and by e14.5, it declines significantly. This decline at e14.5 coincides with the cessation of mitosis in spinal cord neurons, suggesting that GATA3 plays a role in their maturation and differentiation during this stage (George et al. 1994).

According to Kala et al., consistent with the dorsal localization of GATA3‐positive cells in the hindbrain—likely corresponding to neurons in the raphe nuclei—both GATA2 and GATA3 TFs have been shown to play a crucial role in the differentiation of specific neuronal subtypes, particularly serotonergic and glutamatergic populations, within the dorsal raphe (Kala et al. 2009). This structure forms part of a system that extends along the midline of the brainstem, from rostral to caudal, and plays a pivotal role in modulating motor activity and locomotion. In the study, they highlight how both early precursors for vesicular glutamate transporter‐3 (Vglut3) glutamatergic neurons, located in the central dorsal raphe, and serotonin transporter (Sert) serotonergic neurons originate from NK2 homeobox 2 (Nkx2‐2) progenitors. Despite their common origin, these precursors arise in spatially distinct regions and diverge early in development. This needs the influence of different TFs to guide their differentiation into their respective fates. It is noteworthy that GATA2 acts upstream of GATA3, serving as a cell fate selector for both populations. In contrast, GATA3 plays a critical role in the differentiation of Sert+ precursors and in establishing the serotonergic identity of Vglut3+ precursors. Accordantly, the absence of GATA3 has been demonstrated to result in the abnormal development of serotonergic neurons in the caudal raphe nuclei, which is associated with a reduction in locomotor activity. Lack of GATA3 may also result in disruption to cytoarchitectural organization of serotonergic neurons in the caudal raphe nuclei, potentially affecting their motor control functions (Kala et al. 2009).

Another TF from the GATA family that has gained significant attention in recent research is GATA4 (Suzuki 2011). Although GATA4 is well known for its critical role in regulating gene expression during development and maintaining normal physiological functions in various tissues (Holtzinger and Evans 2005), particularly the heart (Tomita‐Mitchell et al. 2007), its expression has also been detected in both neurons and glial cells of the embryonic and adult CNS, either in rodents or in humans (Nawaito et al. 2024). In the brain, GATA4 plays a key role in regulating normal cell growth, specifically by preventing astrocytes from becoming cancerous through the control of cell proliferation and apoptosis (Agnihotri et al. 2011). Notably, the loss of GATA4 has been linked to tumor development, particularly in glioblastoma (Agnihotri et al. 2009). Additional analyses have demonstrated the transcriptional regulatory interaction between GATA4 and GATA6 specifically in newborn murine astrocytes, thereby suggesting their involvement in the later stages of glioma development. However, this cross‐regulation is absent in adult human astrocytes and glioma cell lines (Kamnasaran and Guha 2005).

**FIGURE 2:**
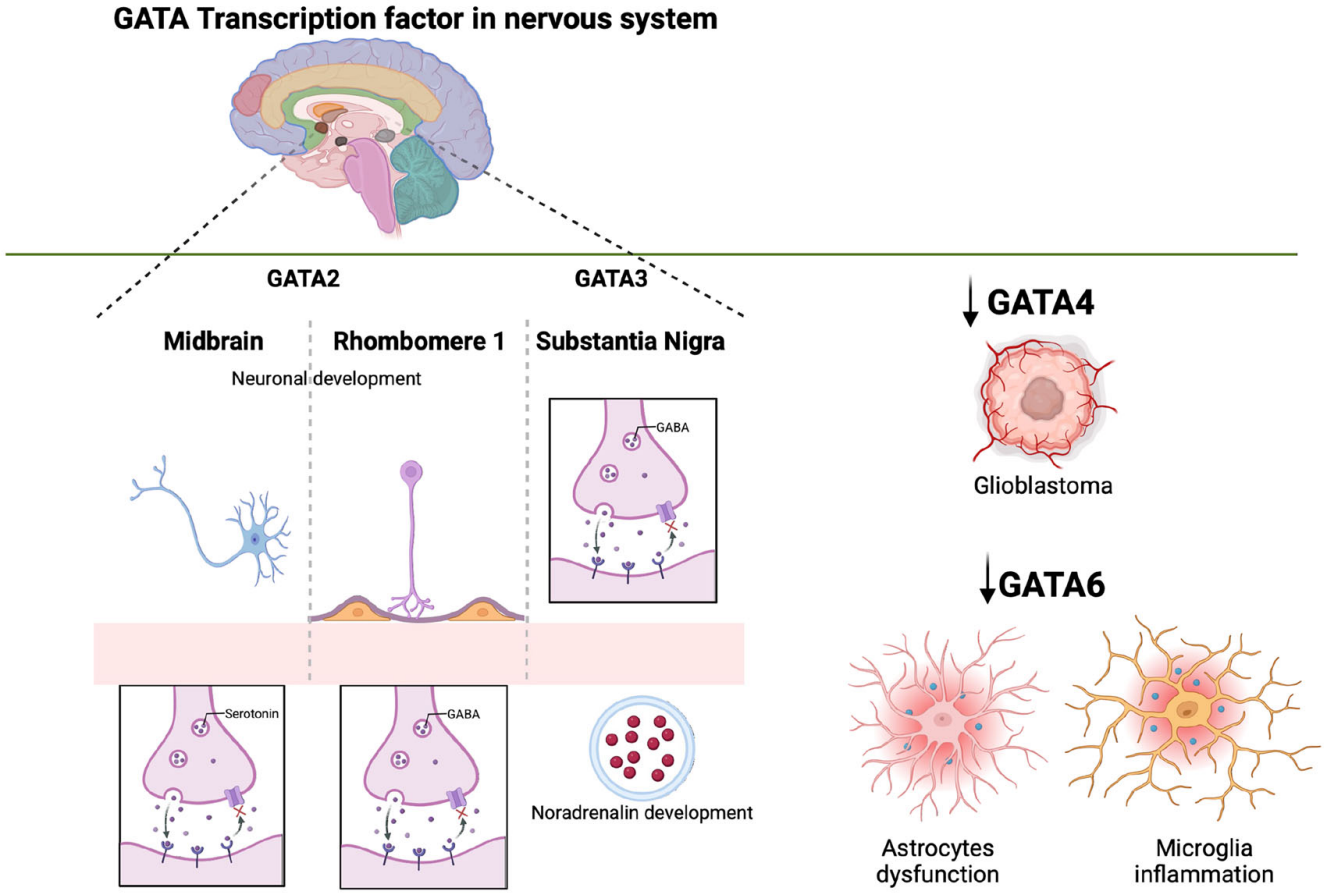
**Implication of GATA transcription factor in the nervous system**. In the central nervous system GATA2 transcription factor is implicated in neuronal differentiation, regulating processes such as the development of serotonergic neurons in the midbrain raphe and GABAergic neurons in the early rhombomeres. GATA3 was observed in some GABAergic neurons and other neuronal subtypes in the *substantia nigra* pars reticulata, where it also regulates the expression of tyrosine hydroxylase (catalyzing the key reaction for the biosynthesis of catecholamines) in the neurons of *pars compacta*. Its absence causes norepinephrine deficiency and embryonic lethality. GATA4 is involved in the control of cell growth and malignancy in astrocytes. Its loss is associated with brain tumors such as glioblastoma. GATA6 is present in neurons, astrocytes, and other nervous cells; it is involved in pro‐inflammatory processes and astrocyte dysfunction in diseases such as spinal muscular atrophy (SMA). *Source*: Created in BioRender (https://BioRender.com/a16e905).

As stated by Kamnasaran et al., GATA6 is expressed in both the adult mouse and human brain. Specifically, with a 2.2 kb transcript indicating its active role in the CNS. Further cellular analysis revealed its presence in the nucleus of various cell types, including neurons, astrocytes, choroid plexus ependymal cells, and endothelial cells (Kamnasaran and Guha 2005). In the context of spinal muscular atrophy (SMA), the reduction of inflammation and microglial phagocytosis of affected astrocytes, following GATA6 knockdown, highlights the central role of GATA6‐driven astrocytic dysfunction in SMA and its role in stimulating pro‐inflammatory pathways and neurotoxicity (Allison et al. 2022). Figure 2 summarizes the role of GATA TF in neuronal development and differentiation.

## 4 The Pivotal Role of GATA Switch in Erythropoiesis and Its Potential Bridging Role With Neurodevelopment

GATA1 belongs to a family of GATA TFs (GATA1‐6) characterized by two zinc finger regions with a well‐conserved architecture reflected in the partial redundancy between the functions of the various members (GATA1‐3) (Cantor and Orkin 2001).

Although GATA family members perform different functions, they do not possess structure‐specific functions, but they are determined by a complex gene regulation during hematopoiesis (Ferreira et al. 2007).

In both mice and humans, *GATA1* is located on the X chromosome and encodes for a TF required for the development of all the hematopoietic cells of many lineages (Kozma et al. 2010; Yu et al. 2002). In erythropoiesis, the “master of regulator” GATA1 plays a central role together with EKLF in both primitive and definitive erythropoiesis (Fujiwara et al. 2004). The GATA1 protein exerts its transcriptional functions by binding, in combination with other TFs/regulatory proteins, regions that regulate the expression of many specific erythroid genes, including the receptor for EPO growth factor and the *GATA1* gene itself (Cantor and Orkin 2001).

GATA1 lineage commitment during hematopoiesis depends on the different target genes’ modulation through the direct binding to the GATA boxes (A/T GATA A/G) in their regulatory regions. GATA1 expression level positively correlates with cell differentiation (Gutiérrez et al. 2020; Gregory et al. 1999): Its expression starts in the common myeloid progenitor stage and increases upon differentiation into ProE (Suzuki et al. 2009, 2003).

Besides GATA1, GATA2 is also expressed in HSCs and HSPCs starting from the early hematopoiesis occurring in the AGM (Suzuki et al. 2006; Kobayashi‐Osaki et al. 2005; Tsai and Orkin 1997). As for all the GATA TF family, both GATA1 and GATA2 recognize similar GATA boxes. Moreover, these two TFs share the same binding sites, resulting in a GATA replacement during hemopoietic cell differentiation (Bresnick et al. 2010). *GATA1* and *GATA2* overlap in function in primitive erythropoiesis, as demonstrated by analysis of double‐knockout embryos (Fujiwara et al. 2004). Consistent with HSC differentiation into erythroid cells, *GATA1* and *GATA2* gene expression profiles have revealed a dynamic transition (Ohneda and Yamamoto 2002), defined as “*GATA* factor switching” (Kaneko et al. 2010).

**FIGURE 3:**
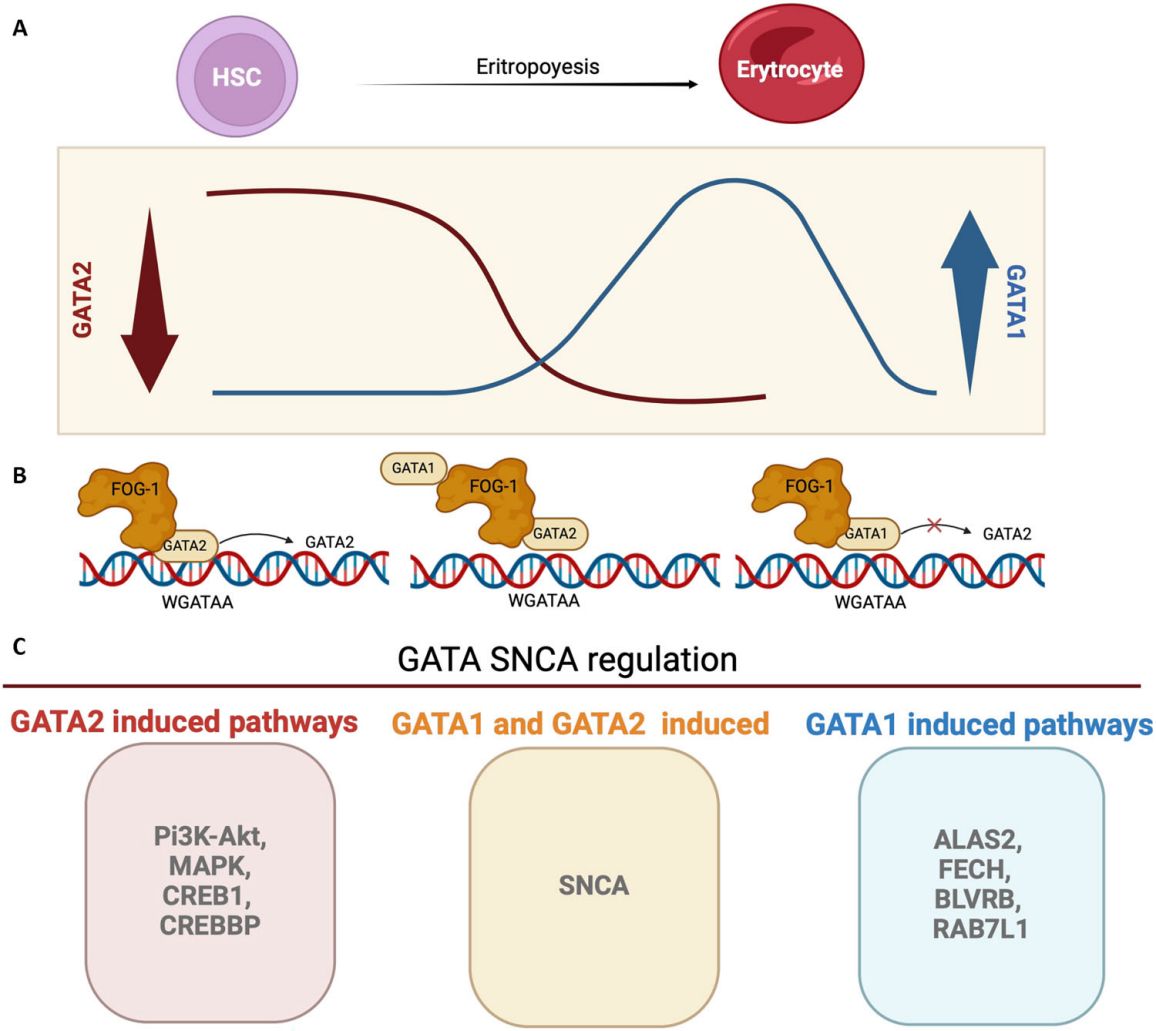
**The transcriptional regulators of erythropoiesis and their role in α‐synuclein regulation**. (A) GATA1 and GATA2 transcription factors (TFs) show dynamic changes in their expression profiles, and these patterns are reciprocal during erythroid differentiation. GATA2 is predominantly expressed in the early stages of erythropoiesis, in hematopoietic stem cells and multipotent progenitors. GATA2 also maintains proliferation and stem potential. The GATA1 TF is progressively expressed at later stages, starting with the most committed progenitors such as erythroid‐committed progenitors. It becomes predominant during the terminal stages, controlling the maturation and differentiation of red blood cells. (B) In the process of erythropoiesis, in the early stages of erythroblast development, GATA2 and Friend of GATA 1 (FOG1) are found together on chromatin sites. As the level of GATA1 increases, this factor replaces GATA2 with the help of Friend of GATA1, which facilitates the switch (GATA switch). (C) The SNCA gene, which encodes for α‐synuclein, is regulated by GATA1 and GATA2. GATA2 induced pathways of the phosphatidylinositol 3‐kinase‐protein kinase B (Pi3K‐Akt), mitogen‐activated protein kinase (MAPK), CAMP responsive element binding protein 1 (CREB), and CREB binding protein (CREBBP). GATA1 induced pathways of delta‐aminolevulinate synthase 2 (ALAS2), ferrochelatase (FECH), biliverdin reductase B (BLVRB), and Rab‐7‐like protein 1 (RAB7L1). *Source*: Created in BioRender (https://BioRender.com/r93y410).

The GATA sites of *GATA2* gene are located at −77, −3.9, −2.8, −1.8, and at +9.5 kb from the transcription start site of the IS exon. The *GATA2* gene expression is supported by multiple *cis*‐acting elements that exhibit distinct properties (Snow et al. 2010). However, when modifications in the *cis*‐ elements at −77 or +9.5 kb occur, they lead to hematologic abnormalities that are not compatible with life (Gao et al. 2013; Marks 2012). On the other hand, *GATA1* gene transcription initiates in the IT (distal testis) and IE (proximal hematopoiesis) promoters (Onodera, Takahashi et al. 1997; Onodera, Yomogida, et al. 1997). The hematopoietic regulatory domain of *GATA1* (G1HRD) consists of one site located −3.9 kb upstream IE in both humans and mice (Nishimura et al. 2000) (Figure 3A,B).

The *GATA2/GATA1* switching was demonstrated by *GATA1* knock‐down mice harboring a reporter transgene driven by *GATA2* gene regulatory region. Through this, it was observed that *GATA2* is expressed in the undifferentiated hematopoietic progenitors (Suzuki et al. 2013).

Immunoprecipitation studies (ChIP), coupled with high‐throughput DNA sequencing, allow the generation of genome‐wide maps of TF binding, clarifying the *GATA1* and *GATA2* relationship (Fujiwara et al. 2009). In particular, GATA motifs that bind both *GATA1* and *GATA2* are divided into distinct categories: GATA motifs preferentially occupied by *GATA2*, those preferentially occupied by *GATA1*, and those equally occupied by *GATA1* and *GATA2* (Suzuki et al. 2013).

GATA‐binding sites for *GATA2* are more expressed in the undifferentiated compartment. Differently, GATA binding sites for the *GATA switching* are driven by *GATA2, GATA1*, and FOG1, which are expressed by the committed erythroid progenitors. Lastly, the *GATA1*‐preferred binding sites are found on terminal erythroid‐affiliated genes (*Hbb, Nfe2*, and *Klf1*) (Kaneko et al. 2010).

Therefore, the *GATA2‐to‐GATA1* switching depends on the chromatin rearrangement: Chromatin accessibility to *GATA1* (defined as *GATA* switching site) begins after the *GATA2* binding. Then, *GATA1* binding activates the gene expression profiles for the erythroid commitment (Moriguchi and Yamamoto 2014; Suzuki et al. 2013). In the preferred *GATA1* binding site, *GATA1* and its partners *(SCL, TCF3, LMO2, and LDB1)* for the erythroid commitment form on a tandem GATA‐E‐box a cooperative pentameric structure, essential for the specific erythroid genes transcription (Wadman et al. 1997). In accordance, the *GATA1* deletion is lethal in embryo mice because of the profound anemia (Pevny et al. 1991).

Among the hypomorphic mutations for *GATA1*, the *GATA1^low^* mutation (also referred to as *GATA1*neo*δ*HS) is represented by the extensive deletion of the hypersensitive HS1 site with a neomycin‐resistant cassette (Ling et al. 2018; McDevitt et al. 1997; Shimizu et al. 2004). The low (∼20%) *GATA1* expressions cause the erythrocytic apoptosis that results in anemia in mice (McDevitt et al. 1997). To compensate for the ineffective erythropoiesis, these mice develop extramedullary hemopoiesis in the spleen (Migliaccio et al. 2009). Together with anemia, *Gata1^low^* mice have impaired megakaryocytic maturation, causing thrombocytopenia (Shivdasani et al. 1997; Tefferi and Vardiman 2008; Centurione et al. 2004; Vyas et al. 1999). The reduced level of GATA1mRNA in adult mice mainly affects the stem/progenitor cells, altering the *GATA2/GATA1* switch that controls the HSPCs proliferation (Bresnick et al. 2010) and favoring their expansion (Ghinassi et al. 2007), together with the *GATA2* increased expression and the inefficient erythroid‐megakaryocytic maturation.

Interestingly, *GATA2* and *GATA1* take part in transcriptional regulatory networks that overlap between neuronal development and RBC maturation, with a growing body of evidence suggesting the biological commonalities between the development of HPCs and neuronal stem cells. For instance, GATA2 may contribute to the balance between proliferation and differentiation of neuronal and glial progenitor cells in the CNS (El Wakil et al. 2006; Tremblay et al. 2018), whereas GATA1 may participate in the differentiation of neuronal and glial lineages. In particular, recent studies have suggested that GATA1 may influence and affect glial cells and that the HSCs express genes specific to the nervous system and responsible for their possible differentiation into oligodendrocytes (Goolsby et al. 2003). Accordantly, a population of neuro‐mesenchymal cells in the AGM HSPC niche was identified in embryo mice (Miladinovic et al. 2024).

Together with GATA1, TCF3 is an important factor that regulates the terminal erythroid differentiations (Chlon and Crispino 2012). TCF3 co‐works for the chromatin remodeling in the *GATA* switching, allowing the GATA1 binding in the *GATA* regulatory sites (Chlon and Crispino 2012; Tremblay et al. 2018). Similarly, in the nervous system, TCF3 has been shown as a modulator of the oligodendrocyte differentiation (Shackleford et al. 2013; Wedel et al. 2020). Thus, even though there is no direct documentation of a functional role of *GATA1* in myelination, this evidence suggests that *GATA1* may be involved in myelin formation and oligodendrocyte differentiation.

## 5 The Role of GATA1 and GATA2 in the Regulation of α‐Syn Expression

α‐syn and GATA TFs are expressed in the nervous system and in the HPSCs. GATA family TFs, particularly GATA1 and GATA2, play an essential role in the regulation of *SNCA*, potentially contributing to the molecular mechanisms underlying synucleinopathies (Figure 3C).

*SNCA* is the major causative gene of dominant familial PD (Cherian and Divya 2020; Guo et al. 2021; Jia et al. 2022), and mutations of this gene are also involved in the pathogenesis of other synucleinopathies (Nussbaum 2018; Orme et al. 2018; Tagliafierro and Chiba‐Falek 2016), as well as of neurodegenerative dementias (Scholz and Cobos 2024). Located on chromosome 4q22.1, *SNCA* comprises 12 exons, with complex regulatory regions in both intronic and exonic sequences, which impact its transcription levels (Clough et al. 2009).

High‐affinity GATA motifs are abundantly distributed across genomes, with ten of them present at the murine *SNCA* gene (Scherzer et al. 2008). *GATA1* predominantly activates *SNCA* transcription in murine erythroid precursor cells by binding a conserved region in the first intron, whereas *GATA2* occupies this site in the absence of *GATA1* (Scherzer et al. 2008). Interestingly, methylation of *SNCA* intron 1 has been shown to repress gene expression, and reduced methylation in this region has been observed in the brains of sporadic PD patients (Schmitt et al. 2024).

Activation of *GATA1* increases SNCA mRNA levels by 62‐fold and α‐syn protein levels by 6.9‐fold. Conversely, *GATA2* silencing in dopaminergic neurons reduces SNCA mRNA and α‐syn protein levels by 28% and 46%, respectively (Scherzer et al. 2008).

In post‐mortem human brains, *GATA2* occupies a specific region in *SNCA* intron 2, as demonstrated by ChIP and EMSA analyses, although these binding sites are not affected by SNPs in PD patients (Brenner et al. 2015). Differences in binding sites described by Brenner et al. and Scherzer et al. may stem from variations in anatomical regions or organisms studied. The potential pathogenetic effects of transcriptional regulation by GATA factors are further supported by studies on the Rep1 simple sequence repeat (SSR) in *SNCA*. This structural variant influences TF binding affinity and has been shown to enhance GATA family binding in PD and DLB patients (Afek et al. 2018). Furthermore, the rs7684318 SNP in *SNCA* exon 4 increases GATA1 binding and alters DNA curvature, strengthening its interaction with TFs (Naushad et al. 2021). These mutations are known risk factors for PD and other synucleinopathies (Chiba‐Falek 2017).

*GATA2* is located in the genomic region most closely associated with genetic predisposition to musical aptitude (Oikkonen et al. 2015). This is partly because *GATA2* plays a key role in cochlear hair cell function and the inferior colliculus, contributing to tonotopic mapping (the brain's ability to process sounds of varying frequencies) (Haugas et al. 2010). Accordantly, the *SNCA* gene, regulated by *GATA2*, is among the most highly expressed genes following music listening and song learning period of zebra finch birds, due to the involvement of α‐syn in dopamine release in the song‐dedicated anterior forebrain pathway (Medina et al. 2022), which links musical abilities to reward mechanisms and dopaminergic signaling (Järvelä 2018; Kanduri et al. 2015; Nair et al. 2021). Additionally, α‐syn is crucial for synaptic plasticity, which is essential for learning and memory processes, including song learning and music learning. Therefore, it is enriched at synaptic terminals in brain regions that exhibit synaptic plasticity, and it is specifically upregulated during periods of synaptic rearrangement related to song acquisition, highlighting its role in the neural mechanisms of musical learning (Cheng et al. 2011).

**FIGURE 4:**
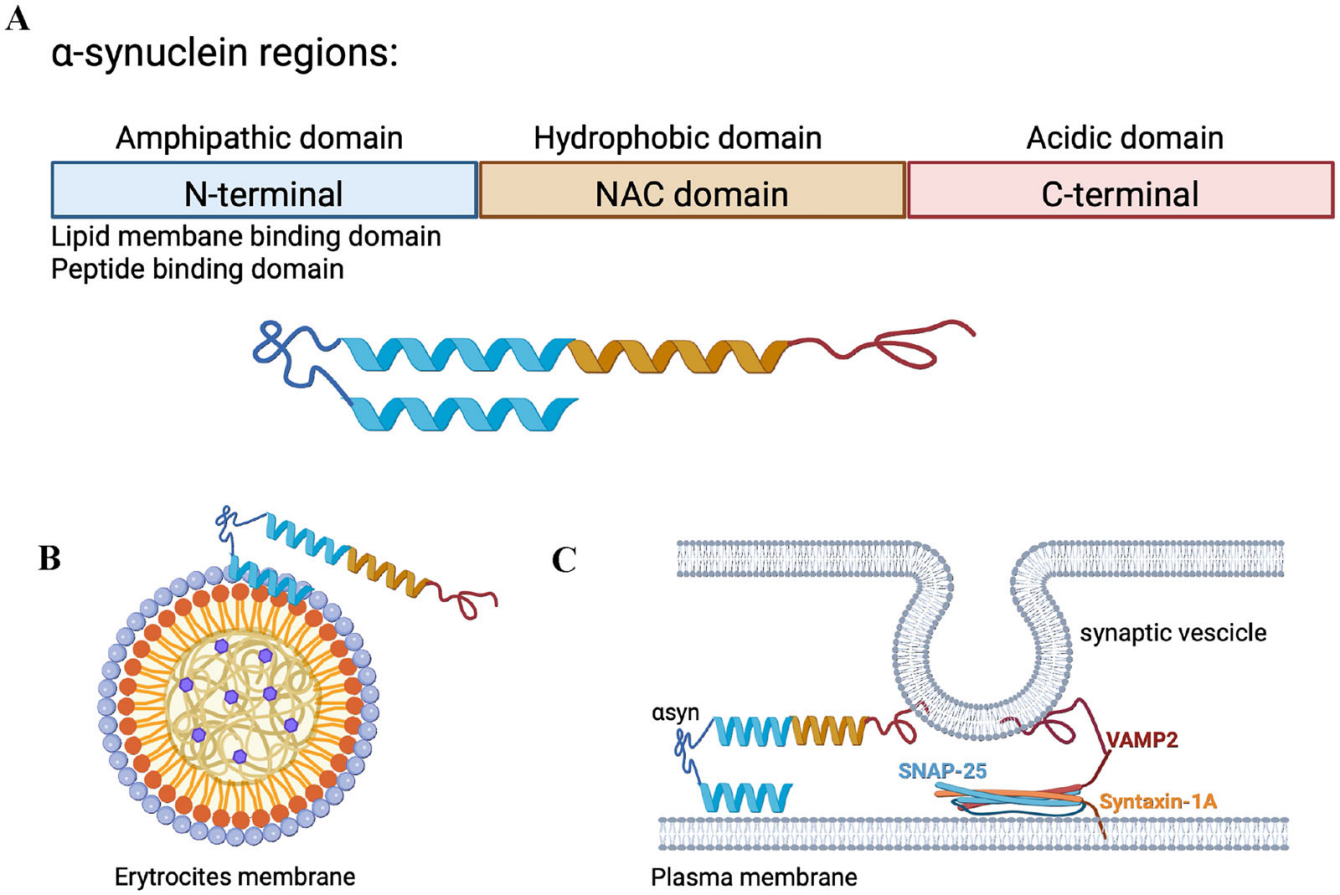
**Figure 4 A‐synuclein and GATA‐1 Transcription factor expression**. (A) The three α‐synuclein regions are represented in different colors. Their properties and interacting sites are indicated. (B) Schematic diagram of N‐terminal region of α‐synuclein that binding the plasmatic membrane of erythrocytes. (C) Schematic diagram of the interaction between α‐synuclein and the SNARE complex (composed of syntaxin‐1A, VAMP2, and SNAP‐25) in membrane interaction. The N‐terminus of α‐synuclein forms two helices to interact with the plasma membrane, whereas the C‐terminus of α‐synuclein interacts with VAMP2. *Source*: Created in BioRender https://BioRender.com/g85a683).

*SNCA* gene is differentially expressed during hematopoiesis and in lymphoma cell lines, either in drosophila or in mammals (Shin et al. 2000). A pivotal biological function of α‐syn has been reported in both erythroid and megakaryocytic lineages, and α‐syn KO mice suffer from mild anemia and platelet abnormalities, along with lymphopenia and immune deficiencies (Xiao et al. 2014). During erythropoiesis, α‐syn is highly expressed by erythroblasts, whereas the expression declines in reticulocytes and erythrocytes (Nakai et al. 2007). More interestingly, the subcellular localization of the protein changes during the different stages of erythroid development, with a prevalent nuclear localization in erythroblasts and a predominant plasma membrane localization in erythrocytes and reticulocytes (Araki et al. 2018). α‐Syn is expressed also in megakaryocytes and platelets. During megakaryocyte differentiation, the protein is continuously upregulated toward the terminal stages so that it becomes abundantly expressed in platelets (Hashimoto et al. 1997). The different subcellular localization and the prevalent nuclear expression in the early stages of both erythroid and megakaryocyte lineages indicate that α‐syn is probably implicated in the transcription of specific genes involved in cell differentiation and maturation. In agreement, anatomical studies have previously reported an extensive nuclear localization of α‐syn in brain and spinal cord regions where neuronal and glial precursor cells are present, such as dentate gyrus (DG), sub‐ventricular zone (SVZ), olfactory bulb and lamina X of the spinal cord (Vivacqua et al. 2011, 2009). In mature blood cells, α‐syn predominantly binds to lipid membranes (Figure 4A,B), increasing membrane mechanical strength and regulating membrane fluidity and curvature (Witt 2013). Moreover, in platelets, α‐syn is associated with the plasma membrane and the membranes of organelles and secretory α‐granules (Hashimoto et al. 1997), where it contributes to the stabilization of the SNARE complex (Reed et al. 2000). In analogy with this, α‐syn enriches synaptic vesicles in neurons where it is the master regulator of membrane curvature and SNARE complex stability (Burré et al. 2014; Cheng et al. 2011) (Figure 4C). Taken together, these data suggest a common function of α‐syn in neuronal and hematopoietic lineages, with a possible role of GATA TFs in their timeframe regulation.

The deficiency of α‐syn in RBCs correlates with reduced oxidative stress, as shown by flow cytometry using a reactive oxygen species (ROS) sensor. This may be linked to decreased expression of inducible nitric oxide synthase (iNOS) and supports the potential role of α‐syn in maintaining ROS and nitric oxide homeostasis (Renella et al. 2014). Interestingly, erythrocytes are emerging as significant players in the pathology of synucleinopathies, highlighting the importance of GATA TF regulation on *SNCA* gene in these cells. Although α‐syn deficiency does not appear to impair early erythroid precursor stages, it has been linked to mild anemia and abnormalities in mature RBCs, including altered morphology and reduced oxygen transport capacity in experimental models (Xiao et al. 2014). Moreover, erythrocytes can contribute to disease mechanisms through extracellular vesicles (EVs). These vesicles, which could contain pathological α‐syn aggregates, can traverse the blood–brain barrier (BBB) and deliver pathologic α‐syn to neural cells, exacerbating neurodegenerative processes and linking peripheral and central α‐syn pathology. Similarly, erythrocyte‐derived EVs can interact with the gastrointestinal tract, suggesting that erythrocytes may play a role in gut‐to‐brain transmission of α‐syn pathology (Yang, Nie et al. 2024). Interestingly, gene analysis in PD indicated a connection between SNCA and iron/Hb metabolism (Santiago and Potashkin 2017), as well as *heme* metabolism, in which α‐syn and GATA1 cooperate in the expression of key genes: *ALAS2*, *FECH*, and *BLVRB* (Scherzer et al. 2008). Moreover, α‐syn can also modulate trafficking of the transferrin/transferrin receptor (Tf/TfR) complex in erythrocytes (Baksi et al. 2016), a mechanism that could be similar in neurons, where altered iron homeostasis has been detected in patients with PD (Sofic et al. 1988).

In neuronal cultures, inhibiting Rho‐GTPase reduces nuclear *GATA2* levels and *SNCA* transcription. *GATA2* forms complexes with serum response factor (SRF) to regulate gene expression, and Rho‐GTPase inhibition induces neurites sprouting, while decreasing α‐syn levels, suggesting a combined role for GATA2 and α‐syn in the expression of cytoskeletal proteins (Zhou et al. 2011). Notably, studies in TLR4^−/−^ mice revealed increased midbrain α‐syn levels unlinked to GATA2 expression. Treatment with 1‐methyl‐4‐phenyl‐1,2,3,6‐tetrahydropyridine (MPTP) did not significantly alter GATA2 protein levels in either mutated or control genotypes (Mariucci et al. 2018). Dopaminergic neuron cultures exposed to 9‐methyl‐β‐carboline (9‐me‐BC) showed a 1.6‐fold increase in GATA2 expression, which interacts with CREB1 and CREBBP to regulate TH synthesis. Despite 9‐me‐BC exposure increased *SNCA* expression by 2.3‐fold, it reduced α‐syn protein levels by 55%, highlighting a complex interplay between transcriptional and post‐transcriptional processes (Polanski et al. 2010).

## 6 GATA TFs and Erythrocyte Abnormalities in Synucleinopathies

Very few studies have investigated the relation of α‐syn with GATA TFs, and above all, experimental evidence about the behavior of GATA TFs during α‐syn aggregation, as occurs in synucleinopathies, is currently unavailable. However, recent evidence supports the involvement of GATA TFs in PD.

Microarray analyses have reported that molecular pathways, including TNF signaling, are related to PD by inducing SMAD family TFs and GATA binding proteins (Tan et al. 2018). Moreover, GATA TF genes have been identified among the crucial regulatory genes involved in PD, together with E2F and E4 promoter‐binding protein (Sun et al. 2018), whereas GATA–bHLH–BNR complexes are involved in Pitx3 genomic regulation of the transcriptional programs in SN and VTA dopaminergic neurons (Bifsha et al. 2017).

GATA1's involvement in PD pathogenesis extends beyond *SNCA*, as evidenced by the rs823144 SNP in the RAB7L1 promoter, which introduces new GATA1 binding sites. *RAB7L1*, part of the *PARK16* locus, has been identified as protective against PD in multiple populations (Gan‐Or 2012). However, studies on five *GATA2* SNPs in a Caucasian PD cohort revealed no significant allele or genotype differences between patients and controls, although the rs3803T variant showed a non‐significant trend toward increased early‐onset PD risk (Kurzawski et al. 2010). Another *SNCA* variant (rs7684318) has been reported as a genetic risk factor for PD. DNAse footprint analyses have revealed that this variant changes the binding of *SNCA* to TFs involved in WNT and Notch signaling, which are in turn implicated in the development of dopaminergic transmission. In particular, rs7684318, characterized by the substitution T > C, increases the binding of RBPJ and GATA‐family TFs and decreases the binding of NKX2 family, SNAI2, SNAI3, DMRT1, HOXA13, HOXB13, HOXC13, HOXD13, WT1, POU4F1, POU4F2, and POU4F3. DNA curvature analyses support the association of this variant with increased binding of GATA1 that contributes to the intensity of DNA curvature peaks and splitting pattern (Naushad et al. 2021).

GATA2 has been implicated in numerous PD‐related molecular pathways. It regulates circRNA networks, including hsa_circRNA_103730 and hsa_circRNA_101275, which are enriched in PI3K–Akt and MAPK signaling pathways, known for their roles in neuroprotection and synaptic plasticity (Xiao et al. 2022). Genetic and transcriptomic analyses have identified *GATA2* as a potential driver gene for rapidly progressing PD (PD‐R), associated with metabolic dysfunction, oxidative stress, neuroinflammation, and dysfunctions in the PI3K–Akt pathway, leading to rapid deterioration of motor and non‐motor functions (Su et al. 2024). Moreover, single‐sequence repeat Rep1 has been associated with the development of PD due to the *cis*‐regulation of *SNCA* gene by the non‐consensus binding mechanism of *GATA2* to *SNCA* (Afek et al. 2018).

Abnormalities in the erythrocytes—such as acanthocytosis—have been detected in patients with different movement disorders and in particular in those affected by chorea acanthocytosis (Ditzel et al. 2023; Peikert et al. 2024). No specific abnormalities have been reported instead in PD patients and synucleinopathies at the early stages. However, in these patients, erythrocytes are more prone to aging and oxidative stress, and membrane abnormalities similar to the cell membrane band‐3 as well as alterations in lipid composition are present at the late stages of the disease (De Franceschi et al. 2004). Moreover, α‐syn aggregation occurs in the erythrocytes of PD patients (Dimoula et al. 2025; Lu et al. 2024; Yang et al. 2024), along with other biochemical abnormalities, including alterations in the iron metabolism (Logroscino et al. 1997), alterations in the expression of genes involved in *heme* metabolism (Scherzer et al. 2008), and formation of Hb–α‐syn complexes either in erythrocytes or in striatal neurons (Yang et al. 2016). Interestingly, the main parts of genes involved in these metabolic processes are mastered by GATA TFs, further bridging erythrocyte biology with synucleinopathies.

### 6.1 Concluding Remarks

GATA TFs are a family of zinc‐finger proteins that play crucial roles in cell differentiation, development, and proliferation across various tissues. These TFs are characterized by their ability to bind to the *GATA* motif within gene promoters, regulating—at different levels—the transcription of target genes. In the nervous tissue, GATA TFs are deeply involved in neurodevelopment, influencing neuronal differentiation, maturation, as well as synaptic plasticity (Balandina et al. 2018). Similarly, in the hematopoietic bone marrow, the timeframe of expression of the GATA TFs contributes to the proliferation of the HPSc and thereafter to the differentiation of both lymphoid and myeloid lineages (Ebihara and Taniuchi 2019; Kato and Igarashi 2019; Rodrigues et al. 2012). The *GATA1/GATA2/GATA3* switch plays a crucial role in the development and differentiation of ectodermal and neuroectodermal tissues, but also in the differentiation of hematopoietic lineages, bridging more closely the biology of hematopoiesis with that of nervous tissue rather than other mesoderm‐derived tissues, where *GATA4/GATA5/GATA6* are mostly employed (Balandina et al. 2018). In accordance, similarities have been discussed in this review between hematopoiesis and neurodevelopment. Similarly to HPSCs, in the context of the nervous system, GATA2 is predominantly expressed in neural progenitor cells, where it promotes the proliferation and the maintenance of stemness. As development progresses and differentiation of different myeloid lineages occurs, GATA1 expression rises, facilitating the transition from progenitor states to fully differentiated blood cells, such as erythroblasts and megakaryocytes (Crispino 2005). The same function seems to be achieved by GATA3 in the nervous system, where this TF rises, promoting the maturation of different neuronal subtypes, including catecholaminergic and GABAergic neurons. This possible switch (*GATA2/GATA3*) may be essential for proper neuronal development, ensuring that the correct types and quantities of neuronal and glial cells are produced. This essential role of *GATA* switch could therefore be crucial for the response of nervous tissue to injury and regeneration, contributing to tissue repair but also to the regulation of genes involved in synaptic plasticity.

α‐Syn, a protein primarily known for its role in synucleinopathies, is particularly linked to GATA TFs, either in the nervous tissue or in the erythroid and megakaryocytic lineages. The expression of the *SNCA* gene is extensively regulated by both GATA1 and GATA2, and we argue that similar functions of α‐syn are employed by nervous and hematopoietic cells. α‐Syn is essential for the maintenance of phospholipid membrane curvature (Sanluca et al. 2024), which occurs both in synaptic vesicles and in the biconcave membrane of the erythrocytes. In line with this evidence, α‐syn is significantly involved in synaptic function, where it contributes to neurotransmitter release and synaptic vesicle cycling (Cheng et al. 2011). Moreover, in synucleinopathies, abnormalities of synaptic transmission and re‐arrangement of the SNARE proteins are early pathological events (Garcia‐Reitböck et al. 2010), and interestingly, they can be associated with alterations at the erythrocytic membrane known as acanthocytosis (Schon et al. 2024). GATA TFs are likely involved in the expression of genes that modulate synaptic function, like CREB proteins, MAPK, or PI3K. Thus, a molecular loop may exist in which GATA TFs regulate the expression of α‐syn and other synaptic proteins, either during crucial developmental windows or during synaptic plasticity in the course of nervous injuries. Disruptions in this putative molecular loop could contribute to the pathogenesis of neurodegenerative diseases. Furthermore, in the context of synucleinopathies, where the aggregation of α‐syn is the key pathological feature, dysregulation of GATA TF activity may exacerbate the toxic events associated with misfolded and aggregated α‐syn. In fact, if GATA factors are unable to properly regulate protective gene expression in the presence of altered α‐syn levels, neurons may become increasingly susceptible to degeneration. Additionally, the interplay between GATA TFs and α‐syn may reflect on neuroinflammatory processes associated with α‐syn pathology. Neuroinflammation can significantly impact neuronal survival and function, and GATA TFs are known to play roles in the inflammatory response in both the nervous system and the bone marrow (Hwang et al. 2022; Liu et al. 2022). The activation of glial cells in response to α‐syn aggregates can lead to the release of pro‐inflammatory cytokines, influencing neuronal biology and contributing to disease progression. The mechanism of GATA TFs in modulating these inflammatory responses could further link their activity with the neurodevelopmental roles of α‐syn in synaptic formation and pruning (Calabresi et al. 2023), further highlighting that disruption of the interplay between GATA TFs and α‐syn may constitute a starring actor in neuronal injury and repair.

In conclusion, the interaction among GATA TFs, α‐syn, and neurobiology discloses a complex regulatory network essential for neurodevelopment, hematopoiesis, neuronal repair, and synaptogenesis. By influencing neuronal differentiation, synaptic plasticity, and neuroinflammation, GATA factors may play a pivotal role in the physiological and pathological functions of α‐syn. Understanding these interactions could pave the way for novel therapeutic strategies targeting both transcriptional regulation and α‐syn‐related pathways in neurodegenerative diseases.

## Author Contributions

**Francesco Bellomi and Claudia Caturano**: conceptualization, literature searching, writing of the first draft, brain immunofluorescence preparation, confocal analysis. **Viola Velardi**: writing the first draft, figures preparation and editing, bone marrow immunofluorescence. **Romina Mancinelli and Simone Carotti**: review of the first draft. **Giorgio Vivacqua**: conceptualization, writing the first draft, review of the first draft for specific intellectual concepts. **Francesca Arciprete**: conceptualization, literature searching, writing the first draft, figures preparation, confocal analysis, editing of the first draft. **Maria Zingariello**: conceptualization, critical review of the first draft and the figures, supervision.

## Conflicts of Interest

The authors declare no conflicts of interest.

## Data Availability

Data sharing is not applicable to this article, as no datasets were generated or analyzed during the current study.
